# Aspects of bovine herpesvirus 1 and bovine viral diarrhoea virus herd-level seroprevalence and vaccination in dairy and beef herds in Northern Ireland

**DOI:** 10.1186/2046-0481-67-18

**Published:** 2014-08-15

**Authors:** D J Bosco Cowley, David A Graham, Maria Guelbenzu, Michael L Doherty, Simon J More

**Affiliations:** 1 MSD Animal Health, Red Oak North, South County Business Park, Leopardstown, Dublin 18, Ireland; 2 Animal Health Ireland, Main Street, Carrick-on-Shannon, Co. Leitrim, Ireland; 3 Agri-food and Biosciences Institute, Stoney Road, Stormont, BT43SD, Belfast Northern Ireland; 4 UCD School of Veterinary Medicine, University College Dublin, Belfield, Dublin 4, Ireland; 5 Centre of Veterinary Epidemiology and Risk Analysis, School of Agriculture, Food Science and Veterinary Medicine, University College Dublin, Belfield, Dublin 4, Ireland

## Abstract

**Background:**

Infections with bovine herpesvirus 1 (BoHV-1) and bovine viral diarrhoea (BVD) virus cause diseases of cattle with a worldwide distribution. The primary objective of the present study was to describe aspects of herd-level BoHV-1 and BVDV seroprevalence (based on testing of pooled sera) and control on farms in Northern Ireland, including vaccine usage.

An indirect antibody ELISA test (SVANOVA, Biotech AB, Uppsala, Sweden) was applied to serum pools which were constructed from serum samples taken for a cross-sectional study of a convenience sample of 500 Northern Irish dairy and beef cow herds in 2010, for which vaccination status was determined by telephone survey. The herd-level seroprevalence of BoHV-1 and BVDV in Northern Ireland was estimated in non-vaccinating herds and associations between possible risk factors (herd type and herd size (quartiles)) and herd-level prevalence were determined using chi-squared analysis.

**Results:**

The herd-level seroprevalence (of BoHV-1 and BVDV) in non-vaccinating herds was 77.3% (95% CI: 73.6–80.9%) and 98.4% (95% CI: 97.3–99.5%) respectively in the cross-sectional study. A significant difference existed in BoHV-1 herd-level seroprevalence between dairy and beef herds (74.7% vs 86.5% respectively; p < 0.02) though not for BVDV seroprevalence (98.5% vs 98.3% respectively; p > 0.91). A significant association was found between herd size (quartiles) and herd-level classification for BoHV-1 herd-level seroprevalence based on cut-off percentage positivity (COPP) (p < 0.01) while no such association was found for BVDV (p = 0.22).

15.5% and 23.8% of farmers used BoHV-1 and BVDV vaccines, respectively. BoHV-1 vaccine was used in 30% of dairy herds and in 11% of beef herds, while BVDV vaccine was used in 46% and 16% of dairy and beef herds, respectively.

**Conclusions:**

The results from this study indicate that the true herd-level seroprevalences to bovine herpesvirus 1 and bovine virus diarrhoea virus in non-vaccinating herds in Northern Northern Ireland are 77.3% (95% CI: 73.6–80.9%) and 98.4% (95% CI: 97.3–99.5%), respectively. The present study will assist in guiding regional policy development and establish a baseline against which the progress of current and future control and eradication programmes can be measured.

## Background

Bovine herpesvirus-1 (BoHV-1) and bovine viral diarrhoea virus (BVDV) cause infectious diseases of cattle with a worldwide distribution [1,2]. A number of Member States within the European Union (EU) have either successfully eradicated these infections or are currently implementing voluntary or compulsory programmes. Herd-level antibody prevalence of each infection shows a wide variation between countries. Strategies for their control and eradication have been previously reviewed [1,3].

In Northern Ireland, limited information is available regarding BoHV-1 infection, albeit from a biased subset of outbreaks [4]. While more information is available regarding seroprevalence of BVDV, this research was restricted to dairy herds. Approximately 90% of those herds were classified with higher grouping for seropositivity, while 5.4% were positive for viral antigen [5]. Furthermore it is some time since this work was conducted. As yet, herd-level prevalence has not been evaluated for either for beef or dairy herds for BoHV-1 or for beef herds for BVDV, and data are not available concerning strategies (including vaccination) being used to control these infections in Northern Ireland. An understanding of the prevalence of these diseases and vaccine usage in their control is necessary for designing and implementing effective national control measures. A number of Member States within the European Union (EU) are considered free of BoHV-1 including Denmark, Germany (the Federal State of Bavaria), Italy (the Province of Bolzano), Austria, Finland, and Sweden [6] while Norway and Switzerland are also considered free [7]. Other countries have EU accredited eradication programmes for BoHV-1 in place (Czech Republic, Germany (all regions, except the Federal State of Bavaria), and Italy (the Autonomous Region of Friuli Venezia Giulia, the Autonomous Province of Trento) [6]. Several other European countries have national control programmes that are not yet accredited by EU legislation. Similarly for BVDV eradication, programmes have been completed or are well-advanced in Scandinavia, Austria and Switzerland. Compulsory national programmes are also underway in Germany and Republic of Ireland and regional eradication programmes have been implemented in France and in the UK (Scotland, Shetland, Orkney and Northern Ireland) [8].

This study describes aspects of BoHV-1 and BVDV infections and their control on farms in Northern Ireland, including herd-level seroprevalence (based on pooled sera) and vaccine usage.

## Methods

### Sample collection

#### Pooled serum

As part of the national statutory brucellosis eradication scheme, serum samples are collected annually from all eligible animals (female bovines and entire bulls aged 12 months or over) in all cattle herds in Northern Ireland and tested by the Agri-food and Biosciences Institute (AFBI). A sample of these herds was selected for the current study. Two herd types were defined in this study–beef (containing >66% beef breed animals) and dairy (containing >66% dairy breed animals). Small herds were excluded (herds <10 breeding cattle). The aim was to select a total sample size of 500 to allow for selection of sufficient herds based on herd-level Se and Sp of tests when used on serum pools and a herd level prevalence of 70% [9].

Samples were received by the AFBI during a ten week period from July to September 2010. Throughout the study period, animals were mostly kept on managed grassland. Permission for inclusion in the study was sought from all selected herdowners. The sera were stored, randomly selected, used to construct serum pools and subsequently analysed using indirect ELISA tests for BoHV-1 and BVDV antibodies (SVANOVA, Biotech AB, Uppsala, Sweden) respectively in AFBI as described in previous studies [9,10]. Herds under brucellosis restrictions are placed under a more intense testing regime but were excluded from the study.

### Vaccine usage survey

A phone survey was used to clarify BoHV-1 and BVDV vaccine usage in each of the study herds. Where relevant, at least three attempts were made to contact each study herd keeper. National vaccine usage data were obtained from GfK Kynetec, an international market research company that gathers data on sales of animal health products [11]. In Northern Ireland, GfK sales data are derived from the main veterinary wholesaler, who distributes all BoHV-1 and BVDV vaccine. Using Microsoft Excel 2003, the data were summarised according to the number of vaccine doses sold by time of application (month) within Northern Ireland.

### Data analysis

#### ***Vaccine usage***

Among the study herds, vaccine usage by seasonality and herd type were evaluated. Vaccine usage in study herds was compared to regional usage data.

#### ***Seroprevalence study***

Pooled serum: The optical density result of each serum pool was recorded. The optimal cut-off PP values (COPP), as determined in earlier studies (by conducting Receiver Operating Characteristics Analysis on replicate results of a preliminary validation study [9,10]), were used to classify each pool as seropositive or negative for BoHV-1 and BVDV, respectively. The prevalences among non-vaccinated herds were separately evaluated. Apparent (and true) herd-level prevalences were calculated from the proportion of positive pools [12]. Associations were evaluated between herd-level seroprevalence classification (based on COPP) and the following parameters: herd type and herd size (quartiles) using a chi-square test.

## Results

### Vaccine usage

**Figure 1:**
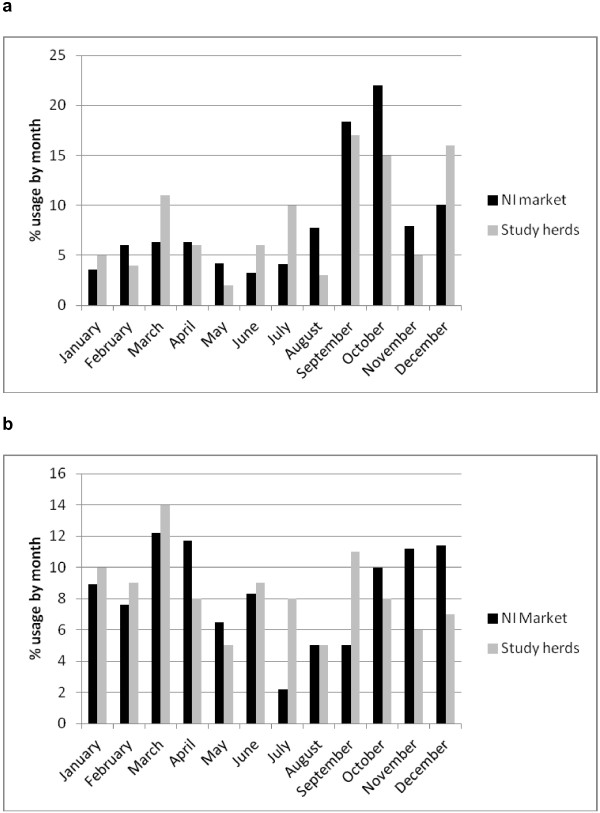
**Monthly vaccine usage on 501 Northern Irish farms and regionally during 2010. a.** shows BoHV-1 vaccine; **b.** shows BVD vaccine.

Interviews were conducted with all of the study herd owners. No information on BoHV-1 and BVDV vaccination history was available for 2.8% and 3.4%, respectively. Data regarding BoHV-1 and BVDV vaccine usage and by herd type are presented in Table 1. Throughout all of Northern Ireland, approximately 550,000 doses of BoHV-1 and 376,000 doses of BVDV vaccine were used in 2010 [11], with a distinctly seasonal pattern of usage. Vaccination in study herds showed a similar seasonal pattern (Figure 1a-b) to overall vaccine usage in Northern Ireland [11].

**Table 1:** Vaccination usage by herd type (herds with no vaccination history excluded)

**Herd type**	**% of herds using vaccine**	
	**BoHV-1**	**BVDV**
**Beef**	**11**	**16**
**Dairy**	**30**	**46**

### Seroprevalence study

#### ***Pooled serum***

Samples from 501 herds were collected during the study period of which 74.6% were classified as beef and 25.4% were dairy, comprising 50,600 animals including 25,800 cows (females > 2 years old). These farms represented just under 2.4% of all beef and dairy herds in Northern Ireland. The exclusion of small herds (containing <10 eligible animals) represented less than 1% of dairy cows, but almost 12% of beef cows [13]. Excluding the 3.4% of herds where no vaccination history was available, herd-level prevalence for BoHV-1 and BVDV in non-vaccinating herds was 77.3% (95% CI: 73.6–80.9%) and 98.4% (95% CI: 97.3–99.5%), respectively. Herd-level classification by herd type is outlined in Table 2 (excluding vaccinating herds and herds with unknown vaccination history). Herd-level seroprevalence values differed significantly between dairy and beef herds for BoHV-1 (74.7% vs 86.5%; p < 0.02) though not for BVDV (98.5% vs 98.3%; p > 0.91). A significant association was found between herd size (quartiles) and herd-level seroprevalence classification for BoHV-1 based on cut-off percentage positivity (COPP) (p < 0.01) while no such association was found for BVDV (p = 0.22) (Table 3).

**Table 2:** Herd level serological classification percentage by herd type

**Herd type**	**Herd level serological classification %**			
	**BoHV-1 negative**	**BoHV-1 positive**	**BVDV negative**	**BVDV positive**
**Beef**	25.3	74.7	1.7	98.3
**Dairy**	13.5	86.5	1.5	98.5

**Table 3:** Herd level serological classification percentage by herd size (quartiles)

**Herd size**	**Herd level serological classification %**			
	**BoHV-1 negative**	**BoHV-1 positive**	**BVDV negative**	**BVDV positive**
**Q1**	37.1	62.9	3.5	96.5
**Q2**	23.1	76.9	1.0	99.0
**Q3**	17.3	82.7	0	100
**Q4**	8.6	91.4	1.9	98.1
	p < 0.01		p = 0.22	

## Discussion and conclusions

Apparent herd-level prevalence of BoHV-1 and BVDV in non-vaccinating herds in Northern Ireland was 77.3% (95% CI: 73.6–80.9%) and 98.4% (95% CI: 97.3–99.5%), respectively.

National herd-level seroprevalence expressed as a percentage of positive herds depends on the selected cut-off, which in turn depends on estimated within-herd prevalence. Cut-off selection and issues relating to sample pooling have been discussed previously [9,10]. Herd classification is based on pooled tests in the seroprevalence study. Because pooling creates complexity, it was assumed that the sensitivity and specificity of the tests when used on the pools was equivalent to these parameters when used on individual sera. However, it is likely that the pooled sensitivity would be lower than single sample sensitivity, especially when within-herd prevalence is low and pool size is large. Individual animals with prolonged seropositivity, along with these other factors, would mitigate any effect on pooled sensitivity. The specificity of a pooled sample should exceed that of an individual sample because of dilution, as discussed previously [9,10].

Prior to the present study, only limited information was available on herd-level prevalence of BoHV-1 or BVDV in the Northern Irish bovine herd, or on vaccine usage at farm level. Herd-level seroprevalences found in the present study are similar to recent findings in the Republic of Ireland [9,10], though comparisons may not be viable as there are significant differences in the source of animals and the regulatory framework between these regions.

Control measures within the current bovine brucellosis control programme in Northern Ireland include annual testing of all herds except some dairy herds which are sampled biennially. Herd testing is coordinated by the local divisional veterinary offices and performed within three months of the overdue date ensuring that the progress of the overall sampling programme is distributed evenly across Northern Ireland. Sampling bias could have arisen due to the convenience sampling model employed in this study rather than true randomized sampling. However, randomization would not suit the model for brucellosis testing implemented in Northern Ireland and as this scheme provided a ready source of samples, this methodology provided the most efficient and cost-effective means of collection of samples from a large number of herds in a short time.

The distinctly seasonal usage of BVDV vaccine (spring and autumn/winter peaks with lower amounts in summer) reflects the calving pattern of the Northern Irish herd, suggesting that most vaccine is used prior to breeding as per manufacturers’ recommendations. Most BoHV-1 vaccine is used in the autumn suggesting usage prior to winter housing. Conventional and marker BoHV-1 vaccines are currently marketed. However the licensed duration of immunity of all BoHV-1 vaccines is six months. Lack of compliance with licensed recommendations may have an impact on the success of vaccination in control. The majority of vaccine is used in dairy herds (Table 1), despite herd-level seroprevalence being higher in dairy herds than beef for BoHV-1 and similar in both herd types for BVDV. Herd level seroprevalence was also found to be significantly higher in larger herds for BoHV-1 though not for BVDV. The latter result is likely to be because herd-level seroprevalence is almost 100%, which precludes differentiation between positive and negative herds on the basis of size. Recall bias could have arisen in this study among farmers surveyed. Vaccine brand used was determined to minimize recall bias and confusion with vaccines used to control other diseases.

Economic losses from BoHV-1 and BVDV infections have been reviewed previously [14,15]. Northern Ireland has a cattle population comprising approximately 281,000 dairy cows and 258,000 beef cows. Significant differences exist in farm size evolution between these two sectors in the 10 year period from 2000 onwards. The average dairy herd size has increased from 54 cows to 77 cows during this period while the average beef herd size has declined from 19 to 17 cows [16]. As a result, significant disease control challenges lie ahead for the dairy sector in Northern Ireland. Current BoHV-1 controls are limited almost exclusively to vaccination, a strategy which, if used alone, is unlikely to have a significant impact in an area of high herd-level prevalence [1]. There are currently no plans to eradicate BoHV-1 in Northern Ireland. A move to prohibit the authorization and use of conventional (non-marker) BoHV-1 vaccines would be a useful initial step towards implementing an eradication scheme at some point in the future considering the current high herd level prevalence of this disease and vaccine usage [1]. A voluntary programme to identify and eliminate animals persistently infected (PI) with BVD virus, through tissue tagging of calves, began in January 2013 with the goal of progressing to a compulsory programme in 2014. This programme mirrors that already in place in the Republic of Ireland, from which lessons may be learned [17]. Additional measures such as certification of herds and implementation of biosecurity measures may be required to make any impact on herd-level seroprevalence for these diseases in Northern Ireland considering the current high levels. Knowledge of seroprevalence is necessary for the design and implementation of effective concerted national control and eradication measures. Furthermore, efforts to increase awareness of these diseases and encourage implementation of cost-effective controls, including screening, elimination of PI animals, vaccination and biosecure practices will help reduce prevalence. However, ultimately these must be coupled with statutory measures to effectively reduce national prevalence. Control of either disease in Northern Ireland will be best effected by cross-border collaboration between the newly formed Animal Health and Welfare Northern Ireland (AHWNI; http://www.animalhealthni.com) and Animal Health Ireland (AHI; http://www.animalhealthireland.ie), industry bodies charged with the national leadership and co-ordination of programmes to address production diseases in Northern Ireland and Republic of Ireland respectively.

## Abbreviations

AFBI: Agri-food and Biosciences Institute, Stormont, Belfast, Northern Ireland; AHI: Animal Health Ireland; AHWNI: Animal Health and Welfare Northern Ireland; BoHV-1: Bovine herpesvirus 1; BVDV: Bovine virus diarrhoea virus; CI: Confidence Interval; COPP: cut-off Percentage Positivity values; EU: European Union; ELISA: Enzyme-linked immunosorbent assay; PP: Percentage Positivity; Se: Sensitivity; Sp: Specificity.

## Competing interests

The corresponding author is an employee of MSD Animal Health (Ireland), however, the company played no role in study design, data collection and analysis, decision to publish, or preparation of the manuscript. The remaining author (s) declare that they have no competing interests.

## Authors’ contributions

DJBC conceived and designed the study, organised the blood sample collection and analysis, designed the owner questionnaires and drafted the manuscript. MG conducted the questionnaires, collected vaccine usage information and conducted the sample analysis. SJM participated in the design and coordination of the study, and DJBC performed the statistical analysis. SJM and DG contributed to the study design and helped to draft the manuscript. All authors read and approved the final manuscript.

## Authors’ information

DJBC is a Master of Veterinary Medicine and works as Technical Manager, MSD Animal Health (Ireland). SJM is UCD Professor of Veterinary Epidemiology and Risk Analysis and Director of the UCD Centre for Veterinary Epidemiology and Risk Analysis (CVERA). DG is project manager for biosecure diseases, Animal Health Ireland. MG is a Veterinary Research Officer with AFBI.
